# Supplementary material for: Comparison of clonal architecture between primary and immunodeficient mouse-engrafted acute myeloid leukemia cells
Source: Nat Commun. 2022 Mar 25;13:1624. doi: 10.1038/s41467-022-29304-6 (PMC8956585; doi:10.1038/s41467-022-29304-6)
Supplement: Supplementary file 1 — Supplementary Information [file 41467_2022_29304_MOESM1_ESM.pdf]

Supplementary Information

Comparison of clonal architecture between primary and immunodeficient mouse-engrafted acute myeloid leukemia cells

Kawashima et al.

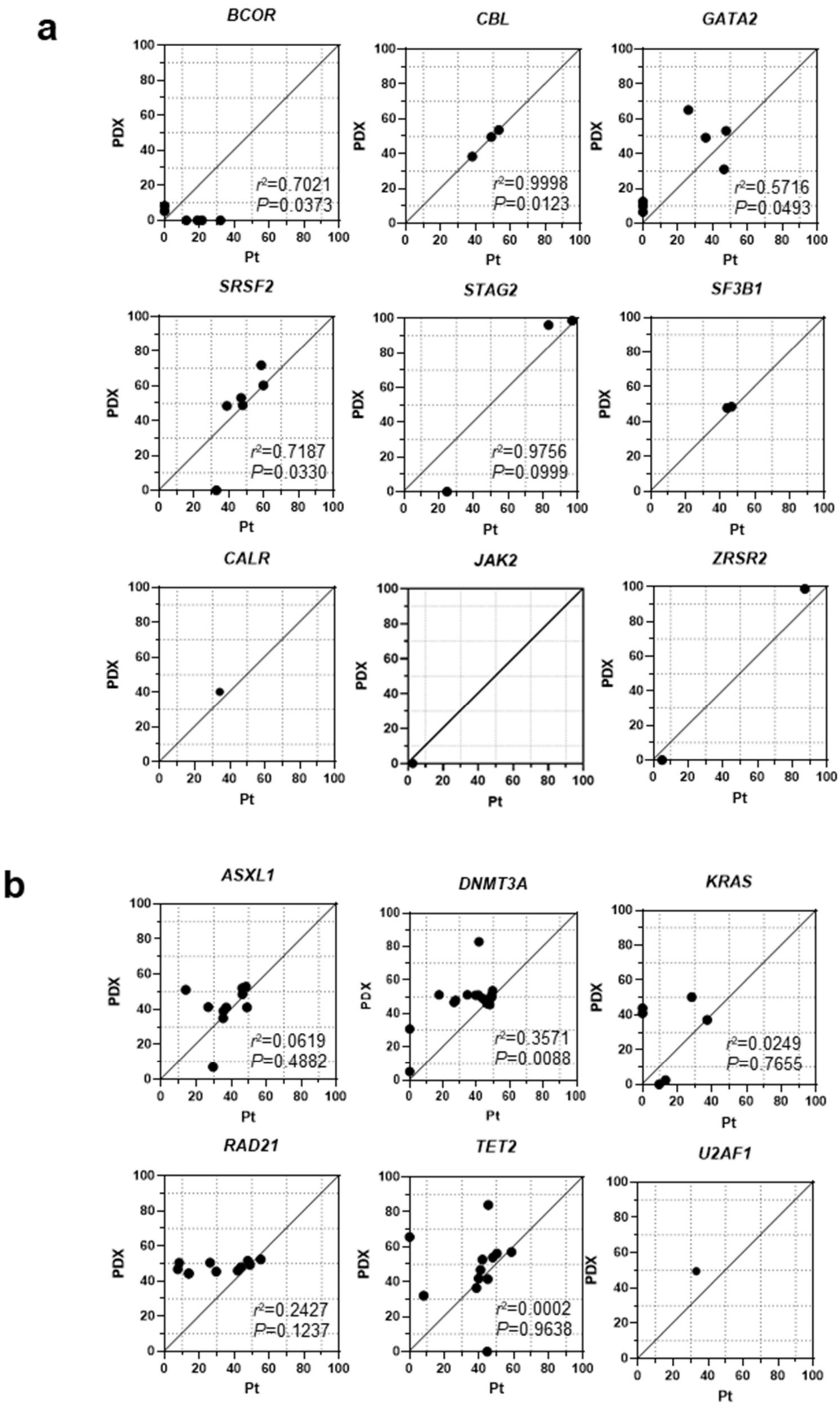

Supplementary Figure 1. VAF changes between primary AML and corresponding PDX models.

Scatter plots of VAF relationships between primary AML cells and P1-2 PDX BM cells in genes with (a) strong correlations and (b) weak correlations.
R^2 values and two-tailed *P*-values calculated using Pearson’s correlation indicate the strength and significance, respectively, of the relationships.

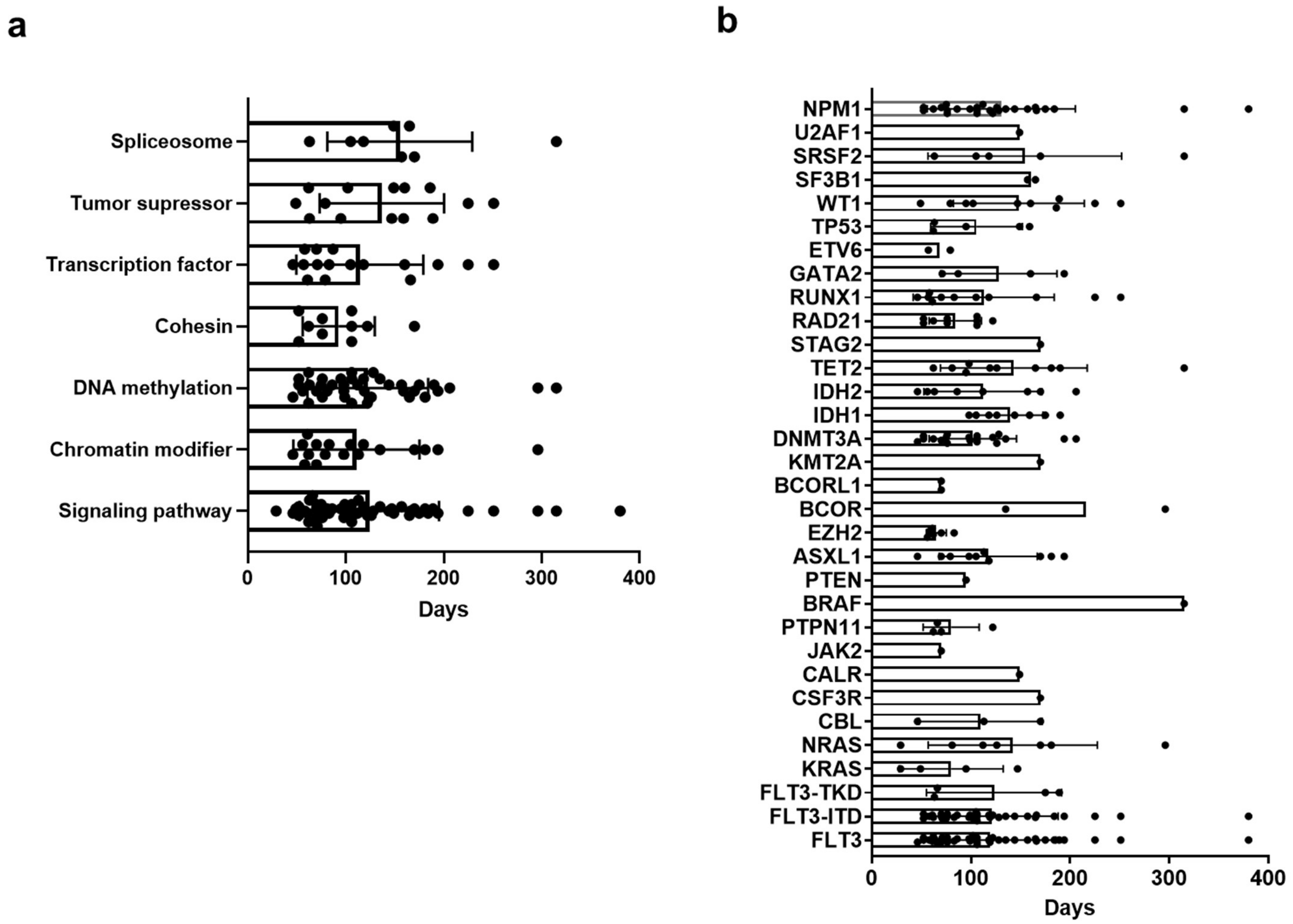

Supplementary Figure 2. Time from transplantation to engraftment according to the mutated genes of transplanted AML cells.

Duration from transplantation to engraftment in PDX according to genetic variants detected in transplanted primary AML cells shown in (a) functional groups (Signaling pathway, *n*=53; Chromatin modifier, *n*=18; DNA methylation, *n*=39; Cohesin, *n*=10; Transcription factor, *n*=16; Tumor suppressor, *n*=14; Spliceosome, *n*=8 biologically independent samples) and (b) individual genes (*FLT3*, *n*=39; *FLT3*-ITD, *n*=34; *FLT3*-TKD, *n*=4; *KRAS*, *n*=4; *NRAS*, *n*=7; *CBL*, *n*=3; *CSF3R*, *n*=1; *CALR*, *n*=1; *JAK2*, *n*=1; *PTPN11*, *n*=4; *BRAF*, *n*=1; *PTEN*, *n*=1; *ASXL1*, *n*=10; *EZH2*, *n*=6; *BCOR*, *n*=2; *BCORL1*, *n*=2; *KMT2A*, *n*=1; *DNMT3A*, *n*=19; *IDH1*, *n*=8; *IDH2*, *n*=8; *TET2*, *n*=10; *STAG2*, *n*=1; *RAD21*, *n*=9; *RUNX1*, *n*=11; *GATA2*, *n*=4; *ETV6*, *n*=2; *TP53*, *n*=5; *WT1*, *n*=10; *SF3B1*, *n*=2; *SRSF2*, *n*=5; *U2AF1*, *n*=1; *NPM1*, *n*=26 biologically independent samples). Data are presented as mean values +/- standard deviations.

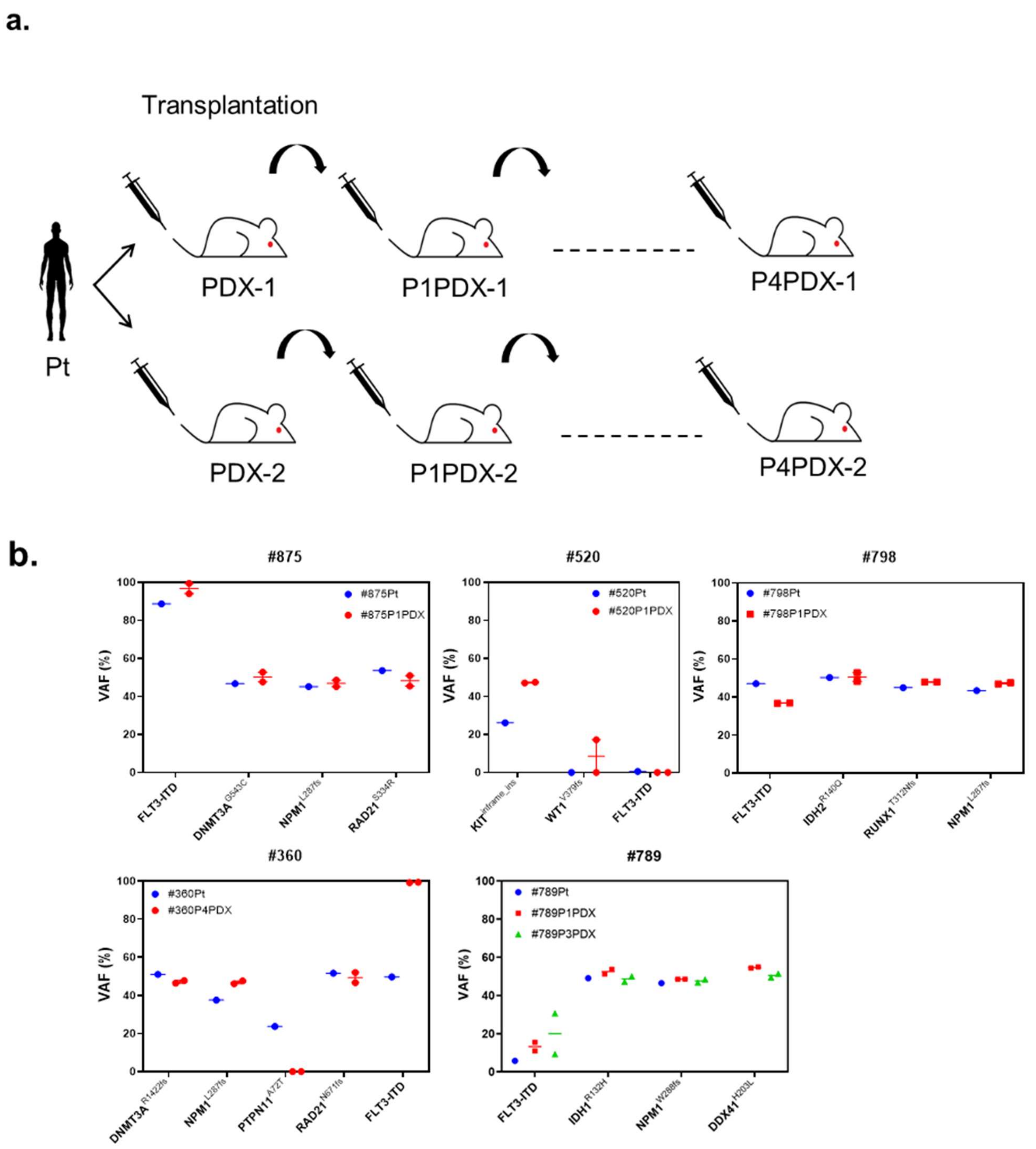

Supplementary Figure 3. VAF changes of somatic mutations in duplicated AML-PDX models.

(a) Experimental design. Fresh mononuclear cells isolated from primary AML cells were intravenously injected into 2 NOG mice and serially transplanted into each line when human CD45+ cells were engrafted.

(b) Dot plots show the VAF of mutated genes in primary AML cells and separately propagated PDX BM cells. Bars indicate the average of the duplicated PDX models.

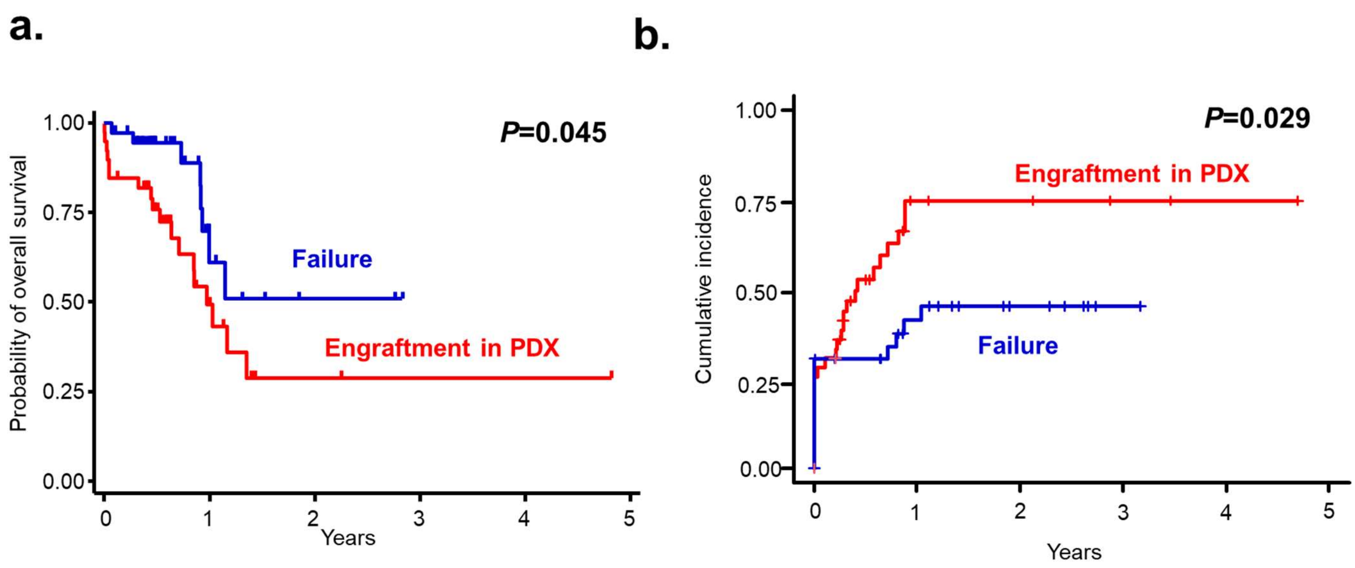

Supplementary Figure 4. Overall survival of AML patients according to the establishment of AML-PDX.

(a) Kaplan-Meier estimates of overall survival and (b) Cumulative incidence of relapse in 76 newly diagnosed AML patients according to the engraftment status in NOG mice. Two-sided P-values were estimated from the Log-rank test in (a) and Gray's test in (b).

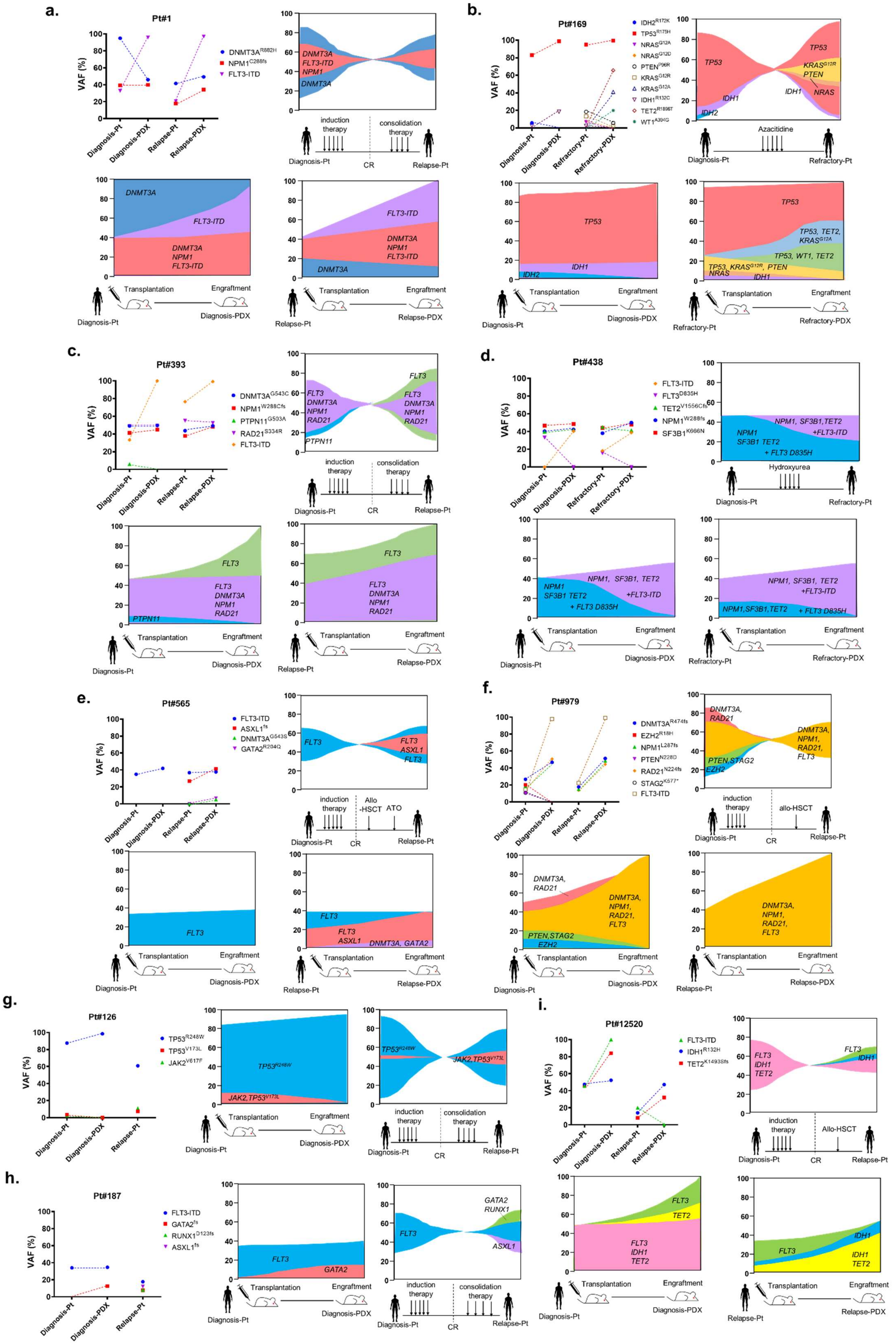

a.
Pt#1
VAF (%)
Diagnosis-Pt
Diagnosis-PDX
Relapse-Pt
Relapse-PDX
induction therapy
consolidation therapy
CR
Transplantation
Engraftment
b.
Pt#169
Azacitidine
Refractory-Pt
Refractory-PDX
c.
Pt#393
d.
Pt#438
Hydroxyurea
e.
Pt#565
Allo-HSCT
ATO
f.
Pt#979
allo-HSCT
g.
Pt#126
h.
Pt#187
i.
Pt#12520
Allo-HSCT

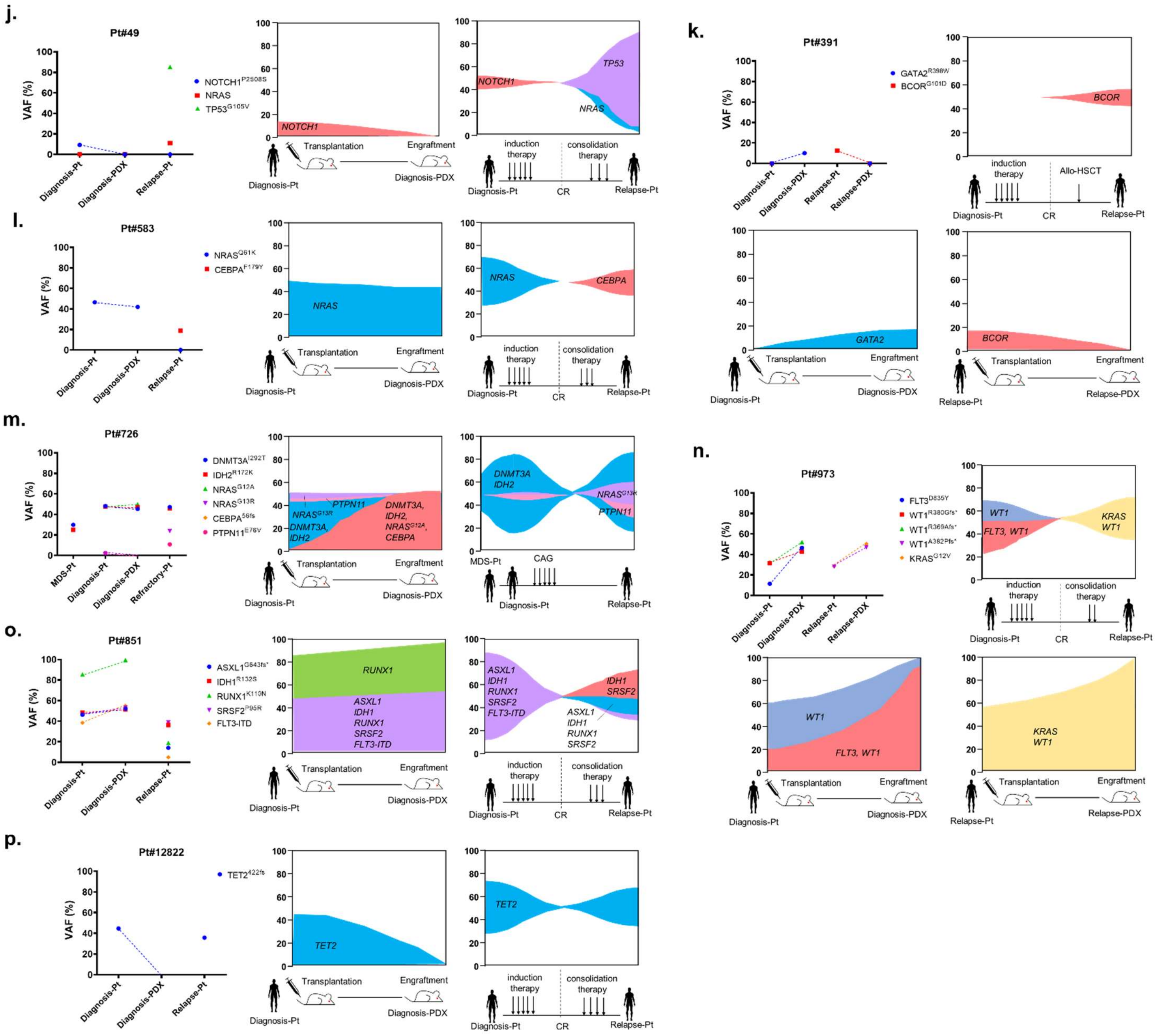

Supplementary Figure 5. Serial mutational spectrum and clonal changes in AML patients and their PDX models.

In each patient, clonal changes in the clinical course and PDX engraftment are shown in the fish plot format. Expanded subclones in PDX generated from AML samples at diagnosis (Diagnosis-PDX) were predictively consistent with the dominant clones at relapse/refractory (Relapse/refractory-Pt) in (a-i) and inconsistent in (j-p).

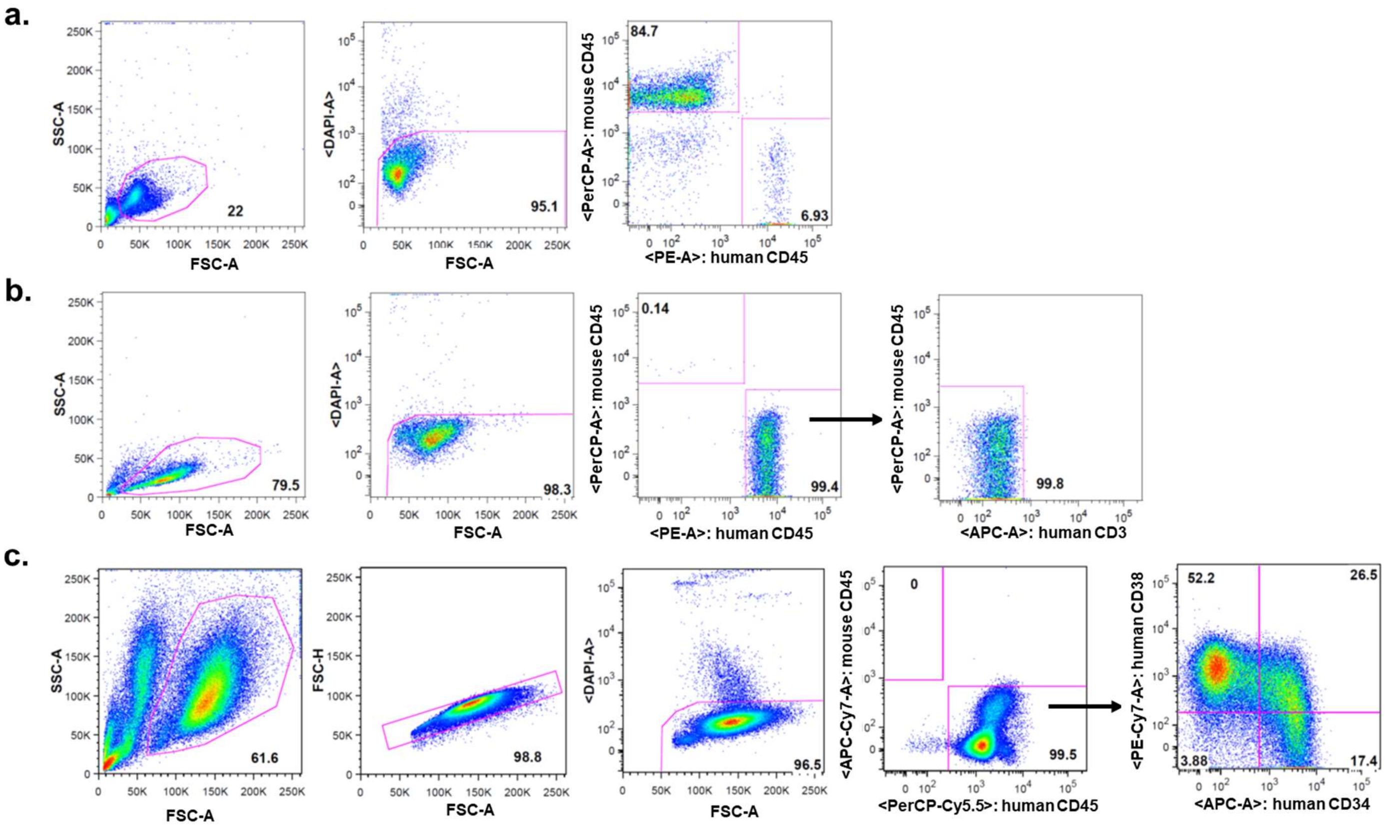

Supplementary Figure 6. Gating strategy for flow cytometry.

AML engraftment was assessed using the percentage of mouseCD45^{-}humanCD45^{+} cells within the live cell compartment through sequential gating in (a) the PB of PDX and that of mouseCD45^{-}humanCD45^{+}CD3^{-} cells in (b) the BM of PDX. (c) The percentages of CD34^{-} and CD38^{-} expression were further assessed in the human CD45^{+} fraction of primary and PDX AML cells.

Supplementary Table 1. Analyzed gene list

ABL1	CEBPA	HRAS	MYD88	SF3B1
ASXL1	CSF3R	IDH1	NOTCH1	SMC1A
ATRX	CUX1	IDH2	NPM1	SMC3
BCOR	DNMT3A	IKZF1	NRAS	SRSF2
BCORL1	ETV6/TEL	JAK2	PDGFRA	STAG2
BRAF	EZH2	JAK3	PHF6	TET2
CALR	FBXW7	KDM6A	PTEN	TP53
CBL	FLT3	KIT	PTPN11	U2AF1
CBLB	GATA1	KRAS	RAD21	WT1
CBLC	GATA2	MLL	RUNX1	ZRSR2
CDKN2A	GNAS	MPL	SETBP1	

Supplementary Table 2. CD34 and CD38 expression in paired primary and PDX AML cells

	Patient				PDX				
UPN	CD34+ CD38-	CD34+ CD38+	CD34- CD38+	CD34- CD38-	CD34+ CD38-	CD34+ CD38+	CD34- CD38+	CD34- CD38-	alteration
7	-	-	+++	+	-	-	++++	+	concordant
49	-	-	++++	-	-	-	++++	-	concordant
137	-	-	++++	+	-	-	++++	+	concordant
187	+	-	-	++++	+	-	-	++++	concordant
469	+	-	-	++++	-	-	-	++++	concordant
606	+	++	++	+	+	+++	++	+	concordant
677	-	+	++++	+	-	-	++++	+	concordant
764	-	-	+++	+	-	-	++	+	concordant
789	-	-	++++	+	-	-	++++	+	concordant
1	+	++	+++	+	-	-	++++	+	discordant
132	++	+	+	+	-	-	+++	+	discordant
252	+	++	++	+	+	+	++	+	discordant
393	+	+	++	+	-	-	+++	+	discordant
396	+	+	+++	+	-	++	++	+	discordant
412	-	-	+	+++	-	-	+++	+	discordant
439	++	++	+	-	-	+	++++	-	discordant
463	++	++	+	-	-	+	+++	-	discordant
520	+	+++	+	-	-	+++	+	-	discordant
573	-	-	+	+++	-	-	++++	+	discordant
583	+	+++	+	-	+	++	++	+	discordant
624	++	+	+	+	-	-	++++	+	discordant
642	-	++	+	+	-	++++	+	-	discordant
664	+	+	+++	+	-	-	++++	-	discordant
696	+	+	++	+	-	-	++++	-	discordant
760	+	++	++	+	+	++	+	+	discordant
786	++++	-	-	-	+++	+	+	+	discordant
798	-	-	+	+++	-	-	+++	++	discordant
979	+	+	++++	+	-	-	++++	+	discordant
999	+	++	+	+	+	+	+++	+	discordant

++++ ≤ 100%, >75% +++ ≤ 75%, >50% ++ ≤ 50%, >25% + ≤ 25%

Supplementary Table 3. Genetic variants engrafted into AML PDX models

Gene	SNV	Frameshift	Inframe indel	Total
FLT3	7	0	35	42
NPM1	0	26	0	26
DNMT3A	11	8	0	19
RUNX1	7	4	2	13
WT1	4	8	0	12
RAD21	3	8	0	11
TET2	3	8	0	11
ASXL1	2	8	0	10
NRAS	9	0	0	9
IDH1	8	0	0	8
IDH2	8	0	0	8
GATA2	6	1	0	7
TP53	6	1	0	7
CEBPA	0	3	2	5
EZH2	5	0	0	5
KRAS	5	0	0	5
SRSF2	3	0	2	5
PTPN11	4	0	0	4
CBL	2	0	1	3
BCOR	2	0	0	2
BCORL1	2	0	0	2
ETV6	1	1	0	2
SF3B1	2	0	0	2
STAG2	1	1	0	2
ABL1	1	0	0	1
BRAF	1	0	0	1
CALR	0	1	0	1
CSF3R	1	0	0	1
KIT	1	0	0	1
KMT2A	1	0	0	1
MPL	1	0	0	1
PTEN	1	0	0	1
SETBP1	1	0	0	1
U2AF1	1	0	0	1
ZRSR2	1	0	0	1
Total	111	78	42	231

Supplementary Table 4. Engraftment Latency

Variables		Median (days)	(range)	*P*-value*
Age	>= 65 years	104	(46-330)	0.863
	< 65 years	111	(27-380)	
Disease	de novo AML	108	(27-380)	0.647
	FAB classification			
	M0	47.5	(46-49)	0.077
	M1	137.5	(98-190)	
	M2	158.5	(46-380)	
	M3	74.5	(34-293)	
	M4	91	(27-362)	
	M5	68	(29-173)	
	M6	113	NA	
	AML with MRC	91	(45-206)	
	tAML from MPN	154.5	(62-187)	
	AL of ambiguous lineage	111.5	(105-118)	
	CML-BC	79	NA	
Graft source	BM	112	(29-362)	0.247
	PB	104.5	(27-380)	
Sample	Diagnosis	138	(9-362)	0.001
	Relapse	102	(45-380)	
	Refractory	76	(27-159)	
Cytogenetic risk	Favorable	104.5	(34-296)	0.935
	Intermediate	112.5	(27-330)	
	Poor	106	(45-362)	
ELN risk category	Favorable	161	(106-330)	0.036
	Intermediate	126	(27-196)	
	Adverse	104	(45-362)	
Infused cell count	>= 5×10^6	106	(27-380)	0.451
	< 5×10^6	128.5	(70-206)	
infused blast %	>= 60%	115.5	(27-362)	0.892
	< 60%	102.5	(45-330)	
	unknown	112	(29-380)	

*Chi-square test for Graft source, Fisher’s exact test for other categorical data and the Mann–Whitney U test for continuous variables.

Supplementary Table 5. Characteristics of patients at diagnosis and relapse/refractory (R/R)

UPN	Diagnosis	FAB	BM Blast at diagnosis (%)	PB Blast at diagnosis (%)	Karyotype at diagnosis	BM Blast at R/R (%)	PB Blast at R/R (%)	Karyotype at R/R	Treatment before R/R	CR duration (days)
1	*de novo* AML	M5b	80	91	Normal	18	0	46,XX,add(3)(q11.2)[2]/46,XX[18]	Consolidation therapy	155
28	*de novo* AML	M2	28.5	2	Normal	NE	86	Normal	Consolidation therapy	39
49	*de novo* AML	M5	85	12.5	49,XY,t(9;11)(p22;q23)	97.5	14	80~90<3n>,XXY,+Y,add(1)(p13),;add(1)(q21),+3,+3,+5,+7,+8,+8,+add(8)(q24),+9,t(9;11)(p22;q23)x2,add(10)(q22),+11,+16,del(17)(p11.2),+18,+19,+20,+22,+3~4mar[cp11]/46,XY[4]	Consolidation therapy	110
126	ET transformed AML		21.5	22	43,XX,del(5)(q?),add(11)(p15),add(12)(p11.2),del(13)(q12q14),del(14)(q22q24),-17, -18,add(21)(q22), -22[1] /43, idem, -add(11), +add(11)(q23)[3] /43, idem, -add(11), +der(11)r(11;?)(p15q23;?)[9]/41~44, XX, del(5), -11, add(12), del(13), del(14), -17, -18, add(21), -22, +r,+0~2mar[cp4]	36	5	43,XX,del(5)(q?),der(11)r(11;?)(p15q23;?),add(12)(p11.2),del(13)(q13q14),del(14)(q22q24),-17,-18,add(21)(q22),-22[11]/43,idem,+add(11)(q23),-der(11)r(11;?),-15,+mar[1]/40~43,XX,del(5),der(11)add(11)(p15)add(11)(q239,add(12),del(13),del(14),-17,-18,add(21),-22[cp6]/46,XX[1]	Consolidation therapy	213
169	MDS overt AML		22	19	44,XX,add(4)(q12),-5,-7, der(12)t(4;12)(q12;p11.2),-15,-18, +der(?)t(?;15)(?;q22), mar [12] /44, idem, add(9)(p22), -13,-17,+2mar [2]/42,idem,add(8)(q24),-16,-20[1]	14.5	9	46,xx, add(2)(q33),add(4)(q12)-5,-7, add(12)(p11.2), der(12)t(4;12)(q12;p11.2), add(4)(q31), -15, -18, der(?)((?;15)(?;q22),+mar[15]/ 44,idem, add(1)(q21),der(2)add(2)(p21)add(2)(q31),+add812)(p11.2)-der(12)t(4;12)(add(4)[5]	Azacitidine	NA
187	*de novo* APL	M3	100	88	46,XY,t(7;14)(q22;q32),t(15;17)(q22;q21)[20]	NE	57	NE	Consolidation therapy	236
266	*de novo* AML	M2	69	87	46,XY,add(9)(q13)[4]/46,XY[16]	50.5	5	46,XY,add(9)(q13)[2]/46,idem,add(1)(p34)[4]/46,XY[14]	Consolidation therapy	321
391	MDS overt AML		32.5	10	46, XX, t(11;19)(q23;q13.1)	58	22	46, XX, add(1)(p36.1),add(4)(q21),del(6)(p21),add(9)(p13),add(10)(q22),t(11;19)(q23;p13.1),?del(12)(p13),-14,add(17)(p11.2),-18,+2mar[18]/46,XX[2]	allo-HSCT	405
393	*de novo* AML	M4	39.2	24	Normal	75	62	46,XX,t(1;15)(p32;q11.2)[3] /46,XX[17]	Consolidation therapy	148
438	*de novo* AML	M4	NE	31	Normal	unknown	unknown	NE	Hydroxyurea	NA
463	*de novo* AML	M4	93.5	96	47XX,+8[16]/47,idem,add(3)(q21)[2]/47,idem,add(3),inv(7)(p15q22)[1]/47,idem,t(8;10)(q24;q24),add(21)(q22)[1]	53.5	71	47,XX,+8[15]/47,idem,t(1;11)(p32;q13)[1]/46,idem,t(1;11),t(3;13)(q26.2;q34),t(5;6)(q13;q27),-18[1]/47,idem,t(3;17)(q27;q11.2)[1]/47,idem,t(15;15)(q11.2;q22)[1]/47,idem,t(19;19)(p13;q13.1-13.3)[1]	Quizartinib	NA
565	secondary APL	M3	NE	60	t(15;17)(q22;q21)	1	6	t(15;17)(q22;q21),?add(19)(p13.1)[6]/46,idem,add(1)(p36.1)[3]/46,idem,-4,add(8)(p11.2),+mar[2]/46,XX[4]	allo-HSCT, ATO	105
583	*de novo* AML	M4	46	35	46,XX,inv(16)(p13;1q22)[20]	20	3	46,XX,inv(16)(p13;1q22)[10]/46,XX[10]	Consolidation therapy	323
606	*de novo* AML	M2	90	88	46,XX,t(7;11)(p15;p15)[20]	90	94	46,XX,t(7;11)(p15;p15)[17/19] 46, idem, t(13;17)(q14;q11.2)[2/19]	Consolidation therapy	263
624	*de novo* AML	M2	84.5	44	46,XX,der(6)t(6;13)(p11;q12),add(13)(q12)[19]/46,XX[1]	NE	70	NE	Consolidation therapy	79
726	MDS overt AML		67	85	46,XY,t(4;21)(q21;q22),add(7)(q22),add(14)(q24)[20]	93	92.5	46,XY,t(4;21)(q21;q22),add(7)(q22),add(14)(q24)[20]	CAG	NA
851	*de novo* acute leukemias of ambiguous lineage, NOS		91	89	Normal	13	2	46, XY, add(1)(p36.2) [5]/ 46, XY [15]	Consolidation therapy	116
973	*de novo* AML	M2	41.5	25	Normal	11.5	23	46,XX,del(9)(q?)[19]/46,XX[1]	Consolidation therapy	105
979	*de novo* AML	M4	30.4	0	47,XX,add(2)(p11.2),add(3)(p25),add(7)(q22),-9,?del(11)(q23),-14,-16,-18,-20,+mar,inc[1]/46,XX[19]	20.4	0	46,XX,t(1;1)(p36.3;q21),add(2)(p.11.2),t(7;14)(q22;q22),add(9)(q34)[2]/46,XX,t(1;12)(p32;p13),add(2),t(7;14)add(9)[1]//46,XY[7]	allo-HSCT	117
12520	*de novo* AML	M1	59.5	58	Normal	28	1	47,XX,del(2)(q?),+6,t(11;12)(q23;q13),add(21)(q22)[2]/46,XY[18]	allo-HSCT	342
12822	*de novo* AML	M4	78.5	34	46,XX,t(5;11)(q33;q13),del(8)(p11.2),t(11;18)(p15;q21.1),add(16)(p13.3)[19]/46,XX[1]	25.5	1	46,XX,t(5;11)(q33;q13),del(8)(p11.2),t(11;18)(p15;q21.1),add(16)(p13.3)[15]/46,idem,t(5;6)(q35;p21)[2]	Consolidation therapy	299

Supplementary Table 6. Primer sequences for fragment analysis and RT-PCR

Fragment analysis for *FLT3*-ITD

Direction	Sequence
Forward	5’- 6-FAM-CAATTTAGGTATGAAAGCCAGC -3’
Reverse	5’- CTTTCAGCATTTTGACGGCAACC -3’

RT-PCR for chimeric gene transcripts

Transcripts	Direction	Sequence
Major *BCR-ABL1*	Forward	5’- GATGCTGACCAACTCGTGTGTG -3’
	Reverse	5’- GGCTTCACACCATTCCCCAT -3’
Minor *BCR-ABL1*	Forward	5’- CAGTCCTTCGACAGCAGCAG -3’
	Reverse	5’- GGCTTCACACCATTCCCCAT -3’
PML-RARA	Forward	5’- TTGCATCACCCAGGGGAAAG -3’
	Reverse	5’- AGGGAGGGCTGGGCACTATC -3’
CBFB-MYH11	Forward	5’- TGATCTCCAAAGACTGGATG -3’
	Reverse	5’- AGTTCCGTCTCATACTCGTG -3’
MLL-AF4	Forward	5’- CAGAATCAGGTCCAGAGCAGAG -3’
	Reverse	5’- GATGACGTTCCTTGCTGAG -3’
MLL-AF9	Forward	5’- GCCTCAGCCACCTACTACAG -3’
	Reverse	5’- ACGATCTGCTGCAGAATGTGTC -3’
MLL-ENL	Forward	5’- GCCTCAGCCACCTACTACAG -3’
	Reverse	5’- GGAGTTGGACGGGCTTGAC -3’
MLL-ELL	Forward	5’- GCCTCAGCCACCTACTACAG -3’
	Reverse	5’- CCGATGTTGGAGAGGTAGAA -3’
