# Supplementary material for: Comparison of clonal architecture between primary and immunodeficient mouse-engrafted acute myeloid leukemia cells
Source: Nat Commun. 2022 Mar 25;13:1624. doi: 10.1038/s41467-022-29304-6 (PMC8956585; doi:10.1038/s41467-022-29304-6)
Supplement: Supplementary file 3 — Reporting Summary [file 41467_2022_29304_MOESM3_ESM.pdf]

nature portfolio

Corresponding author(s): Hitoshi Kiyoi

Last updated by author(s): December 11, 2021

Reporting Summary

Nature Portfolio wishes to improve the reproducibility of the work that we publish. This form provides structure for consistency and transparency in reporting. For further information on Nature Portfolio policies, see our Editorial Policies and the Editorial Policy Checklist.

Statistics

For all statistical analyses, confirm that the following items are present in the figure legend, table legend, main text, or Methods section.

n/a	Confirmed	
☐	☒	The exact sample size (*n*) for each experimental group/condition, given as a discrete number and unit of measurement
☐	☒	A statement on whether measurements were taken from distinct samples or whether the same sample was measured repeatedly
☐	☒	The statistical test(s) used AND whether they are one- or two-sided<br>*Only common tests should be described solely by name; describe more complex techniques in the Methods section.*
☐	☒	A description of all covariates tested
☒	☐	A description of any assumptions or corrections, such as tests of normality and adjustment for multiple comparisons
☐	☒	A full description of the statistical parameters including central tendency (e.g. means) or other basic estimates (e.g. regression coefficient) AND variation (e.g. standard deviation) or associated estimates of uncertainty (e.g. confidence intervals)
☐	☒	For null hypothesis testing, the test statistic (e.g. *F*, *t*, *r*) with confidence intervals, effect sizes, degrees of freedom and *P* value noted<br>*Give P values as exact values whenever suitable.*
☒	☐	For Bayesian analysis, information on the choice of priors and Markov chain Monte Carlo settings
☒	☐	For hierarchical and complex designs, identification of the appropriate level for tests and full reporting of outcomes
☐	☒	Estimates of effect sizes (e.g. Cohen's *d*, Pearson's *r*), indicating how they were calculated

Our web collection on statistics for biologists contains articles on many of the points above.

Software and code

Policy information about availability of computer code

Data collection	Flow cytometry data were collected using FACSAria2 and FACSDiva software v8.0.1 (BD Biosciences). Mutation analysis was performed using Illumina MiSeq sequencer (Illumina) and ABI 3500 Genetic Analyzer and 3500 Series Data Collection Software version 1.0 (Applied Biosystems).
Data analysis	All statistical analyses were performed by Stata version 12 (Stata Corp.) and GraphPad Prism version 6 (GraphPad Software Inc.). Flow cytometry data were analyzed using FlowJo software v8.8.7 (BD Biosciences). Targeted sequencing data were analyzed using Variant Studio software version 3.0 (Illumina). Fragment analysis was performed using GeneMapper analysis software version 4.1 (Applied Biosystems).

For manuscripts utilizing custom algorithms or software that are central to the research but not yet described in published literature, software must be made available to editors and reviewers. We strongly encourage code deposition in a community repository (e.g. GitHub). See the Nature Portfolio guidelines for submitting code & software for further information.

Data

Policy information about availability of data

All manuscripts must include a data availability statement. This statement should provide the following information, where applicable:

- Accession codes, unique identifiers, or web links for publicly available datasets
- A description of any restrictions on data availability
- For clinical datasets or third party data, please ensure that the statement adheres to our policy

The COSMIC database used to define significant somatic mutations can be found at https://cancer.sanger.ac.uk/cosmic. The datasets of targeted sequencing generated and analyzed during the current study are not publicly available due to no consent obtained from the patients for public sharing of their full sequencing data but are available from the corresponding author on reasonable request. Source data are provided with this paper.

Field-specific reporting

Please select the one below that is the best fit for your research. If you are not sure, read the appropriate sections before making your selection.

☒ Life sciences ☐ Behavioural & social sciences ☐ Ecological, evolutionary & environmental sciences

For a reference copy of the document with all sections, see nature.com/documents/nr-reporting-summary-flat.pdf

Life sciences study design

All studies must disclose on these points even when the disclosure is negative.

Sample size	We studied 160 AML patients. Selection of the studied patients was based on the sample availability in our institutions. Statistical methods was not used to assign sample sizes for experiments other than patients study. Sample sizes were based on available data.
Data exclusions	Among 160 patients, 36 AML samples with engraftment and 9 samples with engraftment failure in PDX were excluded from the genetic analysis because the quality of patients' DNA was insufficient or they were derived from different time points of the same patients.
Replication	To assess individual differences between NOG mice, we transplanted primary AML cells with the same cell count into duplicated mice and evaluated discrepancies in engrafted clones using the VAF of each variant in five AML-PDX models. Comprehensive concordance was detected in the VAF of each gene between these duplicated PDX models as shown in Supplementary Figure 3.
Randomization	This is not relevant to our study. Patients and PDXs were allocated to Engraftment/Failure groups retrospectively.
Blinding	Investigators were blinded to Engraftment vs Failure groups allocation during the collection and analysis of patients' data.

Reporting for specific materials, systems and methods

We require information from authors about some types of materials, experimental systems and methods used in many studies. Here, indicate whether each material, system or method listed is relevant to your study. If you are not sure if a list item applies to your research, read the appropriate section before selecting a response.

Materials & experimental systems

n/a	Involved in the study	
☐	☒	Antibodies
☒	☐	Eukaryotic cell lines
☒	☐	Palaeontology and archaeology
☐	☒	Animals and other organisms
☐	☒	Human research participants
☒	☐	Clinical data
☒	☐	Dual use research of concern

Methods

n/a	Involved in the study	
☒	☐	ChIP-seq
☐	☒	Flow cytometry
☒	☐	MRI-based neuroimaging

Antibodies

Antibodies used	anti-mouse CD45-APC/Cy7 (BioLegend, Cat#103115, clone 30-F11, dilution 1:100), anti-human CD45-PerCP/Cy5.5 (BioLegend, Cat#304027, clone HI30, dilution 1:100), anti-human CD45-PE (BD, Cat#555483, clone HI30, dilution 1:100), anti-human CD34-APC (BD, Cat#340441, clone 8G12, dilution 1:100), and anti-human CD38-PE/Cy7 antibodies (BD , Cat#335790, clone HB7, dilution 1:100), anti-human CD3-APC (BD, Cat#555335, clone UCHT1, dilution 1:100)
Validation	No customized antibodies were used. All antibodies used for flowcytometry were from commercial sources and they were purchased from BioLegend and BD. Validation data are available on the manufacturers' web sites and data sheets.<br><br>anti-mouse CD45-APC/Cy7 (BioLegend, Cat#103115, clone 30-F11)<br>https://www.biolegend.com/ja-jp/products/apc-cyanine7-anti-mouse-cd45-antibody-2530?GroupID=BLG1932<br><br>anti-human CD45-PerCP/Cy5.5 (BioLegend, Cat#304027, clone HI30)<br>https://www.biolegend.com/ja-jp/products/percp-cyanine5-5-anti-human-cd45-antibody-4240?GroupID=BLG5926<br><br>anti-human CD45-PE (BD, Cat#555483, clone HI30)<br>https://www.bdbiosciences.com/ja-jp/products/reagents/flow-cytometry-reagents/research-reagents/single-color-antibodies-ruo/pe-mouse-anti-human-cd45.555483<br><br>anti-human CD34-APC (BD, Cat#340441, clone 8G12)<br>https://www.bdbiosciences.com/en-us/products/reagents/flow-cytometry-reagents/clinical-discovery-research/single-color-antibodies-ruo-gmp/apc-mouse-anti-human-cd34.340441

	anti-human CD38-PE/Cy7 antibodies (BD , Cat#335790, clone HB7)<br>https://www.bdbiosciences.com/ja-jp/products/reagents/flow-cytometry-reagents/clinical-discovery-research/single-color-antibodies-ruo-gmp/pe-cy-7-mouse-anti-human-cd38.335790<br><br>anti-human CD3-APC (BD, Cat#555335, clone UCHT1)<br>https://www.bdbiosciences.com/en-us/products/reagents/flow-cytometry-reagents/research-reagents/single-color-antibodies-ruo/apc-mouse-anti-human-cd3.555335

Animals and other organisms

Policy information about studies involving animals; ARRIVE guidelines recommended for reporting animal research

Laboratory animals	6-week-old, male, NOD/Shi-scid, IL-2Rynull mice (NOG) mice were maintained in a 12-hour dark/light cycle, at a temperature of 23 ±2°C and a humidity of 55±10%.
Wild animals	The study did not involve wild animals.
Field-collected samples	The study did not involve samples collected from the field.
Ethics oversight	All animal procedures were approved by the Institutional Animal Care and Use Committee of Nagoya University (M210583-002) and carried out in accordance with the Regulations on Animal Experiments in Nagoya University.

Note that full information on the approval of the study protocol must also be provided in the manuscript.

Human research participants

Policy information about studies involving human research participants

Population characteristics	Primary bone marrow or peripheral blood samples were obtained from AML patients at diagnosis, relapse or refractory to chemotherapy. Patient characteristics are described in Table 1.
Recruitment	Serial patients diagnosed as AML in our institutions were included. Selection of patients was based on sample availability without any self-selection bias.
Ethics oversight	This study was approved by the Institutional Review Board of Nagoya University, Komaki City Hospital, Ichinomiya Municipal Hospital and Japanese Red Cross Nagoya First Hospital.

Note that full information on the approval of the study protocol must also be provided in the manuscript.

Flow Cytometry

Plots

Confirm that:

- [x] The axis labels state the marker and fluorochrome used (e.g. CD4-FITC).
- [x] The axis scales are clearly visible. Include numbers along axes only for bottom left plot of group (a 'group' is an analysis of identical markers).
- [x] All plots are contour plots with outliers or pseudocolor plots.
- [x] A numerical value for number of cells or percentage (with statistics) is provided.

Methodology

Sample preparation	Mononuclear cells were isolated from the fresh bone marrow or peripheral blood samples of primary acute leukemia patients using Ficoll Paque Plus and lysis buffer to lyse RBC cells. Mouse bone marrow cells were flushed out from femurs and treated with lysis buffer. Mouse peripheral blood was collected from tail vein and treated with lysis buffer.<br>PDX peripheral blood samples were used for the analysis of engraftment.<br>Cryopreserved primary AML cells and PDX bone marrow cells were thawed and applied for CD34/CD38 phenotype analysis.<br>Cells were stained with the indicated antibodies diluted in FACS buffer (PBS- 2%FBS) for 30 minutes at room temperature and in the dark. Next, cells were washed with PBS (1500rpm, 4°C, 5 minutes) and 100ng/ml DAPI was added to exclude dead cells.
Instrument	FACSAria2 (BD Biosciences)
Software	Flowcytometry data were collected using FACSDiva and analyzed using Flowjo.
Cell population abundance	Labelled 0.5-1x10^6 cells
Gating strategy	Primary or PDX AML cells were subsequently gated in by physical parameters FSC-A vs SSC-A, singlets (FSC-A vs FSC-H) and viability using DAPI vs FSC-A. For engraftment analysis, they were gated in human-CD45 vs mouse-CD45, and T cell engraftment was further assessed using human-CD3 vs mouse-CD45. Human-CD45 positive and mouse-CD45 negative cells

were subsequently gated in human-CD34 vs human-CD38.
The gating strategy is detailed in Supplementary Figure 6.

☒ Tick this box to confirm that a figure exemplifying the gating strategy is provided in the Supplementary Information.
